# Increase in C‐Reactive Protein Predicts Increase in Rate of Bone Mineral Density Loss: The Study of Women's Health Across the Nation

**DOI:** 10.1002/jbm4.10480

**Published:** 2021-03-16

**Authors:** Gail A Greendale, Nicholas J Jackson, Weijuan Han, MeiHua Huang, Jane A Cauley, Carrie Karvonen‐Gutierrez, Arun S Karlamangla

**Affiliations:** ^1^ Department of Medicine, Division of Geriatrics University of California, Los Angeles (UCLA) Los Angeles CA USA; ^2^ Department of Medicine, Division of General Internal Medicine & Health Services Research University of California, Los Angeles (UCLA) Los Angeles CA USA; ^3^ Department of Epidemiology Graduate School of Public Health, University of Pittsburgh Pittsburgh PA USA; ^4^ Department of Epidemiology School of Public Health, University of Michigan Ann Arbor MI USA

**Keywords:** BONE MINERAL DENSITY, C‐REACTIVE PROTEIN, INFLAMMATION, LONGITUDINAL COHORT, MENOPAUSE

## Abstract

This longitudinal cohort study's aim was to detect whether larger increases in C‐reactive protein (CRP) predict greater amounts of subsequent bone loss in women transitioning from premenopause to postmenopause. Participants were initially 42 to 52 years of age and premenopausal or early perimenopausal. The sample included 1431 women who were not using hormone therapy and whose CRP values were not consistent with acute inflammation. Individual fixed effects (IFE) models estimated the association of log_2_ CRP with subsequent bone mineral density (BMD) decline rate, adjusted for menopause transition (MT) stage (1: premenopausal or early perimenopausal; 2: late perimenopausal or early postmenopausal; or 3: late postmenopausal), body mass index, diabetes, smoking, alcohol, bone active medications, and anti‐inflammatory medications. BMD decline at both the lumbar spine (LS) and femoral neck (FN) was faster for observations made in MT stage 2 than that during other stages (all *p* < .001). In adjusted IFE models, MT stage modified the relation between increase in CRP and BMD decline rate (interaction *p* values <.05). Each within‐woman doubling of CRP was associated with a 0.09% faster yearly decline in FN BMD in MT stages 1 (*p* = .006) and 3 (*p* = .03), and 0.10% faster decline in LS BMD in MT stage 3 only (*p* = .007). Within‐woman increases in CRP in premenopause and early perimenopause and in late postmenopause predict faster BMD decline in the next ~2 years, but the magnitude of CRP's effect is small. © 2021 The Authors. *JBMR Plus* published by Wiley Periodicals LLC on behalf of American Society for Bone and Mineral Research.

## Introduction

In the past decade we witnessed an increasing recognition of the link between the bone and the immune systems, now acknowledged as the field of osteoimmunology.^(^ ^1^, ^2^ ^)^ Not only do the overtly high levels of inflammation that accompany disease states such as rheumatoid arthritis influence bone loss—more modest levels of chronic inflammation (in part, attributed to menopause and/or aging) may also play a role in the genesis of osteoporosis.^(^ ^3^ ^)^ A principal mechanism underlying the inflammation‐osteoporosis link is the direct or indirect (by cytokine secretion) effects of activated T lymphocyte subsets.

Circulating levels of C‐reactive protein (CRP), produced by the liver in response to interleukin 6 (IL‐6) and other cytokines, increase with age in the absence of inflammatory diseases.^(^ ^4^, ^5^, ^6^ ^)^ Greater levels of CRP are associated with lower bone mineral density (BMD) in some, but not all, investigations, the vast majority of which have been cross‐sectional and conducted in older, postmenopausal women and older men.^(^ ^7^, ^8^, ^9^, ^10^, ^11^, ^12^, ^13^, ^14^ ^)^ Longitudinal studies of the relation between change in CRP and change in BMD are lacking.

In women, menopause may promote chronic inflammation, which may in turn be detrimental to BMD. During the menopause transition (MT), the loss of estrogen's modulating influence on inflammatory T cells may unleash cytokine production, as suggested by numerous in vitro and animal experiments.^(^ ^15^ ^)^ For example, estradiol (E2) inhibits the nuclear transmigration of nuclear factor kappa B (NFκB) and subsequent NFκB activation of the IL‐6 gene.^(^ ^4^ ^)^ Murine models of menopause support the menopause‐inflammation‐bone loss pathway.^(^ ^16^ ^)^ Therefore, circulating levels of inflammatory compounds may become elevated during the MT and postmenopause, when E2 levels drop dramatically and reach a new, lower steady state.^(^ ^17^ ^)^

The overarching goal of this analysis was to discern whether increases in CRP predict greater amounts of subsequent bone loss in women transitioning from premenopause to postmenopause. To address this question, we used longitudinal data from the Study of Women's Health Across the Nation (SWAN), a US, community‐based cohort of women experiencing the MT. Specifically, we aimed to: (i) quantify the longitudinal relation between within‐woman change in CRP and ensuing within‐woman change in BMD decline rate; and (ii) if we identified a relation between change in CRP and change in BMD decline rate, discern whether that association varied by MT stage.

## Subjects and Methods

### Study sample

**Fig 1:**
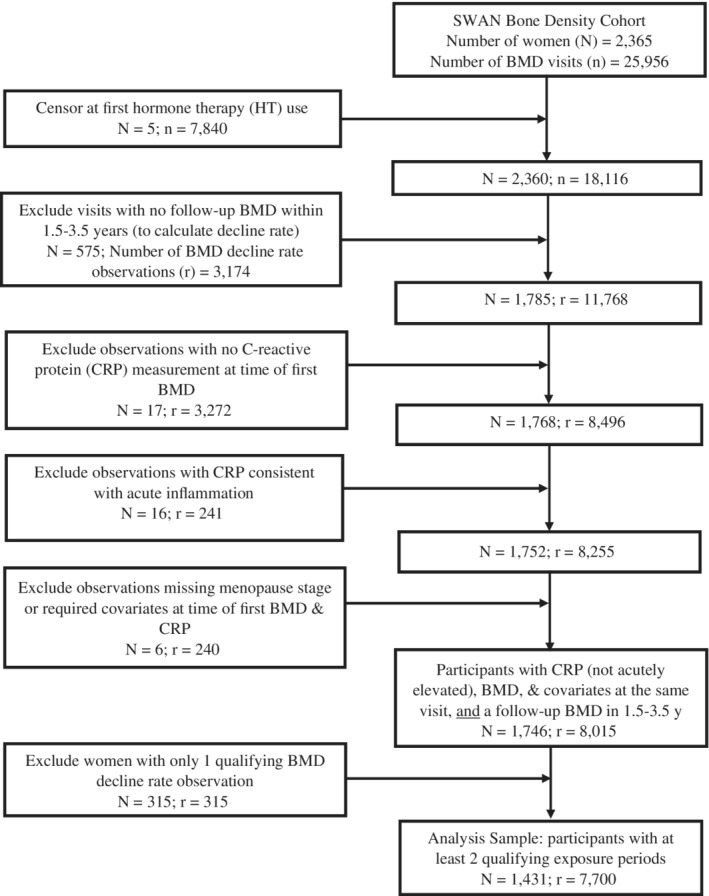
Derivation of the analysis sample. Participants are from the SWAN Bone Cohort. The analysis sample excluded participants using HT at study outset and censored observations from those who initiated HT use during follow‐up. For inclusion, a woman had to have at least two qualifying exposure periods (for rationale, please see Subjects and Methods). A qualifying exposure period consisted of a pair of SWAN visits. At the first of the pair, CRP was measured (and was not judged to be indicative of acute inflammation, see Subjects and Methods for details), a BMD was done, and all required covariates were available. The second half of the pair consisted of a subsequent visit, which occurred between 1.5 and 3.5 years later, and at which a follow‐up BMD (at the same measurement site) was assessed. The set of BMDs was used to compute the rate of BMD loss subsequent to the CRP exposure. To be in the analysis sample, participants had to have at least two qualifying exposure periods. Abbreviations: BMD, bone mineral density; CRP, C‐reactive protein; HT, hormone therapy; SWAN, Study of Women's Health Across the Nation.

SWAN is a multi‐site, community‐based, longitudinal cohort study of the MT; initial eligibility criteria were age between 42 and 52 years, intact uterus and at least one ovary, not currently using hormone therapy (HT), at least one menstrual period in the 3 months before screening, and self‐identification as a member of one of five eligible ethnic groups.^(^ ^18^ ^)^ All seven clinical sites enrolled White women, four sites enrolled Black women and the remaining three sites enrolled Chinese, Hispanic, and Japanese women, respectively, for a total of 3302 participants. Two centers did not measure BMD; therefore, 2413 SWAN participants at five SWAN sites were eligible for the bone density cohort. This analysis encompassed data from SWAN baseline through SWAN follow‐up visit 16. The current analysis excluded participants using HT at baseline and censored data at HT initiation in those who initiated it during follow‐up. The dependent variable in our analyses is rate of change in BMD between SWAN visits. To allow inclusion of women who may have missed an interim visit, we allowed the time between visits (for BMD change rate calculation) to span 1.5 to 3.5 years; the median number of years between BMD visits in a change‐rate calculation was 2.0 (interquartile range [IQR], 1.9 to 2.1). The primary predictor in our analyses is CRP. For a BMD change‐rate observation (from a pair of visits) to qualify for inclusion in the analysis, the first visit in the pair had to have a CRP measurement that did not represent acute inflammation (see Data Analysis for criteria for identifying acute inflammation), and covariates available. To maximize causal inference from longitudinal observational data, we used individual fixed effects (IFE) analyses, which focus on the relationship between within‐woman changes in predictor and dependent variable, and removes all confounding by time‐fixed characteristics of the participant (including characteristics that are not measured). Because within‐woman change is known only if predictor and dependent variables are measured at least twice, our analysis sample excluded data from women who had only one qualifying BMD decline rate observation. Figure 1 illustrates the sample derivation. Participants gave written informed consent and each site obtained institutional review board (IRB) approval.

### Outcome: BMD

Hologic instruments measured LS and FN BMD (g/cm^2^) (Hologic, Inc., Waltham, MA, USA). To create a cross‐site calibration, SWAN circulated an anthropomorphic spine phantom. Boston, Detroit, and Los Angeles sites began SWAN with Hologic 4500A models and subsequently upgraded to Hologic Discovery A instruments. Davis and Pittsburgh started with Hologic 2000 models and later upgraded to Hologic 4500A machines. When a site upgraded hardware, it scanned 40 women on both its old and new machines to cross‐calibrate them. A standard quality control program included daily phantom measurements, local site review of all scans, central review of scans that met problem‐flagging criteria and central review of a 5% random sample of scans. Short‐term in vivo measurement variability was 0.014 g/cm^2^ (1.4%) for the LS and 0.016 g/cm^2^ (2.2%) for the FN. Two of the five bone sites could not access dual‐energy X‐ray absorptiometry (DEXA) machines for the entirety of follow‐up, thus the maximum possible number of BMDs per participant varies by SWAN site. As of 2020, at three of the five BMD sites, this potential maximum was 16; for one site, it was 13; and for one site it was 15. The median number of BMDs per participant, incorporating all five BMD sites, is 13 (IQR, 8 to 15; range, 1–16). At the two SWAN sites with maximum potential BMD scans of 13 or 15, the median number of per participant BMDs was 12.

### Primary predictor: high‐sensitivity CRP

SWAN measured CRP (mg/L) at baseline through follow‐up visit 7, and at follow‐ups 9, 10, 12, and 15. Because of the long observation period, CRP assays changed over time (three were used), as did their lower limits of detectability (LLD). Therefore, SWAN cross calibrated all three CRP assays to a single fourth assay, Human High Sensitivity CRP (hs‐CRP) enzyme‐linked immunosorbent assay (ELISA) (R&D Systems, Minneapolis, MN, USA; DCRP00). From baseline to follow‐up visit 7, SWAN used an ultrasensitive rate immunonephelometric method to assay CRP, with an LLD of 0.3 mg/L (Dade‐Behring, Marburg, Germany; BN100). The intraassay coefficient of variation (CV) at CRP concentrations of 0.5 and 22.0 mg/L were 10–12% and 5–7%, respectively. At follow‐up visit 12, we assayed CRP using the ACE UltraWide Range assay, a latex‐enhanced turbidimetric in vitro immunoassay (Alfa Wassermann, West Caldwell, NJ, USA). The LLD was 0.1 mg/L, and the intraassay CV at CRP concentrations of 0.5 and 9.8 mg/L were 5.7–7.0% and 1.2%, respectively. Samples from follow‐up visits 9, 10, and 15 were assayed using a high‐sensitivity immunoassay (Alfa Wassermann, West Caldwell, NJ, USA). The LLD was 0.1 mg/L, and the intraassay CV at CRP concentrations of 0.5 and 9.8 mg/L were 5.7–7.0% and 1.2%, respectively. In 25% of all samples run between baseline and follow‐up 15, CRP was below the LLD of these original three assays. In these instances, a stored sample was retrieved from the SWAN Repository and CRP was measured using the Human High Sensitivity CRP ELISA. The remaining 75% of results (those above the LLD for the three original assays) were calibrated to the high‐sensitivity ELISA by simultaneously assaying 600 paired samples (representing the full range of results from each of the original three assays) and the high‐sensitivity assay. Thus, there were 200 paired samples for each original and high‐sensitivity ELISA. After calibration, correlations between the original three CRP assays and the fourth, high‐sensitivity ELISA were each >0.94.

### Covariates

Age (years), self‐defined race/ethnicity (Black, Chinese, Japanese, White), menstrual bleeding patterns, HT use (yes/no, time varying), diabetes mellitus (yes/no, time varying), current medications (see detailed list later in this paragraph; yes/no, time varying), current calcium or vitamin D supplement use (yes/no, time varying), cigarette use (any/none), and alcohol consumption (abstinent; infrequent, ≤1 drink per week; light to moderate, >1 drink per week and ≤1 per day; heavy, >1 drink per day) were ascertained using standardized questionnaires and interviews at each study visit. Weight (kilograms) and height (meters) were assessed at each visit using calibrated scales and stadiometers. We used menstrual histories, acquired at each visit, to create MT stages (time varying). MT stages include premenopausal (regular menses, unchanged from usual), early perimenopausal (menses within the prior 3 months but less predictable than prior), late perimenopausal (≥3 months but <12 consecutive months of amenorrhea) and postmenopausal (no menses for ≥12 consecutive months). Postmenopause was further divided into early postmenopause and late postmenopause. SWAN defined late postmenopause as starting two visits after the visit at which menopause was identified, to emulate to the Stages of Reproductive Aging Workshop (STRAW) definition of later menopause.^(^ ^19^ ^)^ For this analysis, we combined MT stages: stage 1, consisting of premenopause or early perimenopause; stage 2 comprising late perimenopause and early postmenopause; and stage 3, late postmenopause. These menstrually‐based stages approximate the early transition, the middle transition and the post‐transition.^(^ ^17^, ^20^, ^21^ ^)^ Body mass index (BMI) was calculated ([weight in kilograms/(height in meters)^2^]). For each visit, indicator variables (any/none) captured diabetes diagnosis and use of any of the following: bone beneficial drugs (bisphosphonates, raloxifene, calcitonin, parathyroid hormone, or calcitriol): bone adverse drugs (gonadotropin‐releasing hormone [GnRH] agonists, aromatase inhibitors, chemotherapy, or anti‐seizure medications); anti‐inflammatory medications other than oral steroids (aspirin or non‐steroidal anti‐inflammatories [NSAIDs], oral steroids, supplemental calcium; or vitamin D. Time‐varying covariates also included current smoking (any/none) and alcohol consumption (four categories, described earlier in this paragraph).

### Statistical analysis

#### Classifying acute inflammation

The total number of CRP observations in SWAN was 16,482. If we were to use the customary absolute cut‐point of 10 mg/L as the criterion for exclusion, 2156 observations (13%) would have been eliminated. However, an absolute value of 10 mg/L will discard many observations that probably reflect *chronic* stable inflammation, especially in Black women or those who are obese and could have been a source of biased estimation.^(^ ^22^ ^)^ Therefore, to identify visits in which a CRP measurement likely reflected *acute* inflammation, we used a hierarchical strategy to capture acute elevations in CRP and to avoid removing higher, but stable, values. The strategy uses a multiplier factor, *f*; CRP values that are larger than *f* times a comparator CRP value from the same woman at other study visits are deemed to represent *acute* inflammation. The value of the multiplier, *f*, was chosen by a team of investigators which included clinicians, to allow identification of values that are temporary outliers relative to a participant's CRP at other study visits. The best choice for *f* was determined to depend on the value of the comparator; when the comparator is low, *f* is high; when the comparator is high, *f* is low: *f* = 6 if comparator ≤1; *f* = 6/sqrt (comparator) if comparator >1 but ≤9, and *f* = 2 if comparator ≥9. To allow for up to three CRP values from a participant to be categorized as acute inflammation, we applied a three‐step hierarchical strategy, based on comparing her maximum CRP, the second maximum CRP, the third maximum CRP, and the trimmed mean (the participant's mean CRP value computed excluding the first, second, and third maxima). Step 1: if the third maximum CRP is greater than *f* times trimmed mean, then all three maxima are considered acute inflammation values (end of strategy for that participant). If this condition is not met, continue to step 2. Step 2: if second maximum CRP is greater than *f* times the third maximum CRP, then both the maximum CRP and the second maximum CRP are acute inflammation values (end of strategy for that participant). If this condition is not met, proceed to step 3. Step 3: If the maximum CRP is greater than *f* times the second maximum CRP, then the maximum CRP is an acute inflammation value. We omitted CRP observations that met these criteria for acute inflammation. Of the 16,482 CRP levels measured in the entire SWAN sample, using the hierarchical method, 516 qualified as acute inflammation. Restricted to the 8496 observations in the current analysis sample, the method classified 241 as acute inflammation. In the entire SWAN sample, the median and IQR of the ratio of the “acute” CRP to its comparator was 11.9 (IQR, 8.5 to 19.7) when the comparator was ≤1 mg/dL, and 8.7 (IQR, 5.9 to 12.8) when the comparator was >1 but ≤3 mg/dL. The median and IQR of the CRP values deemed acute inflammation were 20.2 (IQR, 16.4 to 29.2) when the comparator was >3 but ≤9 mg/dL, and 36.4 (IQR, 29.8 to 54.7) when the comparator was >9 mg/dL. Comparing the results of the hierarchical method of identifying acute inflammation to the commonly‐used absolute CRP exclusion value of ≥10 mg/L, 1758 CRP observations that were ≥10 mg/L that would have been excluded by the absolute cut‐point were retained using the hierarchical method (~11% of the SWAN study total). Conversely, 118 CRP observations that were less than 10 mg/L, and would not have been excluded by the absolute cut‐point of 10 mg/L, were eliminated using the hierarchical criteria (~1% of the SWAN study total).

#### Baseline characteristics and change in characteristics over time

The sample characteristics at the study outset were summarized as the mean ± standard deviation (SD) for continuous variables and the number (%) for categorical variables. Study outset refers to the first qualified BMD change rate observation; that is, the first pair of study visits 1.5 to 3.5 years apart, with CRP measured at the first of the two paired visits and BMD measured at both visits (at LS and/or FN). For all variables other than BMD slope (annualized change rate), which was computed from BMD measurements at both visits in the pair, sample characteristics refer to measurements made at the first of the two paired visits. Because the distribution of CRP was non‐normal, we log transformed it (to base 2) for all analyses and summarized it as mean and SD of log_2_(CRP). We used base 2 log transformation to allow interpretation of estimated CRP effects as associations with doubling of CRP. At both the FN and the LS, we calculated BMD slope (or annualized change rate, in % per year) as the percentage increase in BMD from the first to the second paired visit, divided by the time elapsed between the two visits.

We also summarized the within‐women change in sample characteristics from the first qualified BMD change rate observation (henceforth called observation) to follow‐up observations. For continuous variables, we summarized crude change in characteristics as the mean and SD of change from the first observation, over all follow‐up observations. For categorical variables, two quantities that capture change during follow‐up were calculated. First, is “new since baseline”: the number of observations during which women newly reported “yes” (i.e., said “no” at first but subsequently report “yes”). Second is “stopped since baseline”: the number of observations when women newly reported “no” (i.e., “yes” at first but subsequently report “no”).

#### Change in CRP and BMD slope by MT stage

Because the MT is a major driver of BMD decline, we also computed the mean and SD of the within‐woman change in primary predictor (log_2_ CRP) and outcomes (BMD slope at FN and LS) from the first observation to follow up observations, separately for follow‐up observations in each of the three MT stages.^(^ ^20^, ^21^ ^)^ MT stage for each follow‐up observation was determined as the MT stage at the first of the paired visits that make up that observation. In statistical testing for differences in mean across MT stages, we accounted for correlations between repeated observations in the same women using Huber‐White robust estimates of standard errors.

#### Longitudinal associations between CRP and BMD

We used IFE regression to quantify the relation between CRP and BMD slope in the specified interval (1.5–3.5 years) following each CRP measure. BMD slope (percent change per year) was the dependent variable and log_2_ (CRP) at the start of each observation interval was the continuous primary predictor. IFE regression can be interpreted as ordinary linear regression after every variable in the model (dependent variable, primary predictor, and all covariates) has had its participant‐specific mean (over all observations) subtracted from it. Thus, the two distinct advantages of IFE regressions. The first is that it focuses exclusively on within‐woman change, so that the IFE model estimates the relationship of within‐individual change in the primary predictor to within‐individual change in the dependent variable. The second advantage is that participant characteristics and variables that do not change over time do not influence model estimates, thus eliminating *all* confounding by individual‐specific characteristics that do not change over time (i.e., *time‐fixed*). This includes confounding by *time‐fixed* variables that are measured (e.g., race/ethnicity, study site), and those that have or cannot be ascertained (e.g., genetics, family history).^(^ ^23^, ^24^ ^)^ To control for confounding by *time‐varying* covariates, we created a series of nested models. Model 1 included MT stage, BMI, cigarette use, diabetes, and alcohol use. Model 2 covariates additionally included bone‐beneficial medications, bone‐negative medications, oral corticosteroids, and NSAIDs and aspirin. Model 3 added supplemental calcium and vitamin D. We tested for effect modification of the relation between CRP and BMD by MT stage; if we detected interactions, we conducted pairwise‐comparisons. The effect sizes presented quantify the relation between within‐individual changes in the time‐varying predictor and the time‐varying outcome. Because log_2_ CRP was the exposure variable, effect sizes represent the increment in annualized BMD slope per each within‐woman doubling of CRP.

## Results

The study sample consisted of 1431 women with mean ± SD age of 45.6 ± 2.6 years at study outset. The median duration of follow‐up in years, from the first observation (first qualified visit pair) to the last observation (final qualified visit pair) was 10.6 (IQR, 6.1 to 15.3) years. Ethnic/racial groups represented were White (48%), Black (25%), Japanese (14%), and Chinese (13%). Table 1 summarizes the pertinent characteristics of the analysis sample at the initial observation, when 97% of the sample was either premenopausal or early perimenopausal. The mean of log_2_(CRP) over all women's first observations was 0.53, which translates to a geometric mean of raw (not logged) CRP of 1.44 mg/L. Mean values of first on‐study LS and BMD measures were 1.07 g/cm^2^ at the LS and 0.84 g/cm^2^ at the FN. Additionally, the mean values of within‐woman BMD slope during the first observation were −0.23% per year and −0.18% per year at the LS and FN, respectively. (Each observation consists of two SWAN visits; the first supplies a CRP and accompanying BMD and the second supplies a second BMD, which is used to calculate that observation period's BMD rate of change.)

**Table 1:** Characteristics of Analysis Sample at Study Initiation and Change in Time‐Varying Characteristics During Follow‐Up

Characteristic	Initial value^a^ (1431 women)	Change during follow‐up observations^b^ (7700 observations; 1431 initial, 6269 follow‐up)	
Age (years)	45.6 ± 2.6	5.3 ± 3.5	
Body mass index (kg/m^2^)	27.4 ± 6.8	0.87 ± 2.23	
Log_2_CRP (log_2_ mg/L)	0.53 ± 2.07	0.10 ± 1.31	
BMD			
Lumbar spine, g/cm^2^	1.07 ± 0.14	−0.03 ± 0.06	
Femoral neck, g/cm^2^	0.84 ± 0.13	−0.02 ± 0.05	
Initial rate of BMD change^c^			
Lumbar spine (% per year)	−0.23 ± 1.53	−0.65 ± 2.34	
Femoral neck (% per year)	−0.18 ± 1.77	−0.62 ± 2.61	
Race, *n* (%)			
White	686 (47.9)	NA	
Black	364 (25.4)	NA	
Japanese	198 (13.8)	NA	
Chinese	183 (12.8)	NA	
		New since baseline	Stopped since baseline
Menopause transition stage, *n* (%)^d^			
Pre/Early Peri (Stage 1)	1393 (97.3)	0 (0.0)	2577 (41.1)
Late Peri/Early Post (Stage 2)	32 (2.2)	1046 (16.7)	74 (1.2)
Late Post (Stage 3)	6 (0.4)	1605 25.6)	0 (0.0)
Diabetes, *n* (%)	82 (5.7)	238 (3.8)	86 (1.4)
Current smoking (any), *n* (%)	202 (14.1)	79 (1.3)	194 (3.1)
Alcohol use, *n* (%)^e^			
None	787 (55.0)	1456 (23.2)	295 (4.7)
Infrequent	313 (21.9)	609 (9.7)	956 (15.2)
Moderate	262 (18.3)	240 (3.8)	891 (14.2)
Heavy	69 (4.8)	62 (1.0)	225 (3.6)
Supplemental vitamin D, *n* (%)	544 (38.0)	1378 (22.0)	730 (11.6)
Supplemental calcium, *n* (%)	663 (46.3)	1407 (22.4)	711 (11.3)
Bone‐adverse medications, *n* (%)^f^	42 (2.9)	281 (4.5)	80 (1.3)
Bone‐beneficial medications, *n* (%)^g^	1 (0.1)	142 (2.3)	3 (0.0)
Oral corticosteroids, *n* (%)	27 (1.9)	190 (3.0)	77 (1.2)
NSAIDs, *n* (%)	313 (21.9)	1243 (19.8)	551 (8.8)
Aspirin, *n* (%)	104 (7.3)	592 (9.4)	234 (3.7)

Abbreviations: BMD, bone mineral density; CRP, C‐reactive protein; GnRH, gonadotropin‐releasing hormone; NA, not applicable; NSAID, nonsteroidal anti‐inflammatory drug; SD, standard deviation; SWAN, Study of Women's Health Across the Nation.

^a^ Values are means ± SDs of continuous variables and numbers (%) of categorical variables; categorical values shown are numbers responding affirmatively. Initial values are from the first SWAN study visit used in this analysis.

^b^ For continuous variables, we list the mean and SD of change from individual‐specific initial value over all follow‐up study visits. For categorical variables, we list two descriptive quantities of change: the number of observations at which women newly reported “yes” (i.e., were “no” at initial visit and now report “yes”) and the number observations at which women newly report “no” (i.e., were “yes” at initial measure and are now “no”). The number within parentheses is the percentage of 6269 total follow‐up observations that report such changes.

^c^ Initial rate of BMD change computed from the first exposure period, which consists of two SWAN visits. The first SWAN visit supplies a CRP and accompanying BMD and a second SWAN visit (that occurred ~2 years later) supplies the second BMD. These two BMDs are used to calculate the initial exposure period's BMD rate of change. Positive values indicate growth and negative values indicate loss.

^d^ Pre = premenopausal (regular menses, unchanged from usual); early peri = early perimenopausal (menses within the prior 3 months but less predictable than prior); late peri = late perimenopausal (≥3 months but <12 consecutive months of amenorrhea); early post = first 3 visits at which participant is deemed postmenopausal (menopause defined as no menses for ≥12 consecutive months); late post = postmenopausal, subsequent to early postmenopause. For this analysis, we group MT stages into the following categories: stage 1 is premenopause and early perimenopause, stage 2 is late perimenopause and early postmenopause, and stage 3 is late postmenopause.

^e^ Infrequent, ≤1 drink per week; light to moderate, >1 drink per week and ≤1 per day; heavy, >1 drink per day.

^f^ Yes to any of the following: GnRH agonists, aromatase inhibitors, chemotherapy, or anti‐seizure medications.

^g^ Yes to any of the following: bisphosphonates, raloxifene, calcitonin, parathyroid hormone, or calcitriol.

Table 1 also estimates changes in malleable characteristics that occurred during the 6269 follow‐up observations. The median number of observations per woman was six (IQR, 3 to 7). Over all follow‐up observations, mean change in BMD slope was −0.65% and −0.62% per year at the LS and FN, respectively. Mean overall change in log_2_(CRP) was 0.10, which translates to a 7.2% increase in raw (not logged) CRP during the study. However, the standard deviation of change in log_2_(CRP) was 1.31, equivalent to a 2.5‐fold increase. Changes in categorical variables since the initial visit are shown as “new since baseline” and “stopped since baseline”. For example, 2.9% of participants initially reported using bone adverse medications (GnRH agonists, aromatase inhibitors, chemotherapy, or anti‐seizure medications). At 4.5% of the follow‐up observations, women who did not use bone‐adverse medications initially reported newly using them (“new since baseline”). Conversely, at 1.3% of follow‐up observations, women who initially used bone‐adverse medications no longer did so (“stopped since baseline”).

The crude mean of log_2_ CRP over all observations was 0.61 log_2_ mg/L and the geometric mean of the raw (not logged) CRP was 1.53 mg/L (data not tabulated). On average, unadjusted level of CRP increased as MT stage progressed from stage 1 to stage 2, but stabilized in stage 3. During stage 1, mean log_2_ CRP level was 0.51 log_2_ mg/L, corresponding to a geometric mean of 1.42 mg/L. In stage 2, log_2_ CRP averaged 0.85 log_2_ mg/L, a geometric mean of 1.80 mg/L. The stage 3 mean log_2_ CRP was 0.73 log_2_ mg/L, equal to a geometric mean of 1.66 mg/L.

Spanning all observations, crude mean of BMD slope was −0.76% per year at the LS and −0.67% per year at the FN (data not tabulated). From stage 1 to stage 2, LS BMD slope went from −0.61% per year to −1.61% per year. During stage 3, the LS BMD slope slowed to −0.65% per year. LS BMD slope did not differ between stages 1 and 3. Similarly, average FN BMD slope went from −0.45% annually in stage 1 to −1.28% in stage 2. The average stage 3 FN BMD slope slowed to −0.95% yearly.

Table 2 provides the crude mean value of the within‐woman changes in log_2_CRP and BMD slope during each MT stage, relative to the first on‐study values. Within‐woman change in log_2_ CRP was approximately the same in each MT stage, that is, around +0.10, which translates to 7.2% increase in raw CRP (all pairwise *p* values >.77). In contrast, the crude mean of within‐woman change in BMD slope (referenced to the first on‐study BMD slope) varied by MT stage. In stage 1, change in slopes at the LS and FN BMD sites were −0.76% per year and −0.55% per year, and nearly doubled in stage 2. In stage 3, LS BMD within‐woman change was ~0.0%, whereas at the FN, the rate of loss reduced to −0.46%.

**Table 2:** Descriptive Statistics for Within‐Woman Change in CRP and Within‐Woman Change in Annualized Slopes of LS and FN BMD, by MT Stage

MT stage^a^	Change in log_2_ CRP (log mg/L)^b^ (observations) mean ± SD	Change in LS BMD slope (%/y)^c^ (observations) mean ± SD	Change in FN BMD slope (%/y)^c^ (observations) mean ± SD
Stage 1	(3587) **0.10** ± 1.30	(3520) **−0.76** ± 2.14	(3549) **−0.55** ± 2.49
Stage 2	(1068) 0.09 ± 1.28	(1056) **−1.29** ± 2.41	(1044) **−1.06** ± 2.70
Stage 3	(1614) **0.11** ± 1.37	(1585) **0.02** ± 2.57	(1582) **−0.46** ± 2.78
Omnibus *p* (*p* values for differences in mean within‐woman change, by MT stage^d^)			
Stage 1 vs. stage 2	.82	<.001	<.001
Stage 2 vs. stage 3	.77	<.001	<.001
Stage 1 vs. stage 3	.94	<.001	.33

Abbreviations: BMD, bone mineral density; CRP, C‐reactive protein; FN, femoral neck; LS, lumbar spine; MT, menopause transition; SD, standard deviation; SWAN, Study of Women's Health Across the Nation.

^a^ Stage 1 includes premenopause and early perimenopause (premenopause defined as menses unchanged from usual; early perimenopause defined as menses in prior 3 months but less predictable than usual). Stage 2 includes late perimenopause and early postmenopause (late postmenopause defined by at least 3 months but not 12 consecutive months of amenorrhea; early postmenopausal defined as the first 3 visits at which participant is postmenopausal, menopause having been determined by 12 consecutive months of amenorrhea). Stage 3 is late postmenopause, defined as the time subsequent to early postmenopause.

^b^ Arithmetic mean (and SD) of within‐woman change in log_2_ CRP values from baseline visit to follow up exposure period. MT stage used for stratification is the stage at the follow up visit. Mean change shown in bold font if the mean is statistically different from zero (*p* < .05).

^c^ Arithmetic mean (and SD) of change in annualized BMD slope from first exposure period to follow‐up exposure period. The first exposure period consists of two SWAN visits. The first SWAN visit supplies a CRP and accompanying BMD and a second SWAN visit (that occurred ~2 years later) supplies the second BMD (used to calculate that exposure period's BMD rate of change). Each subsequent exposure period consists of a CRP and BMD obtained at a following SWAN visit and second BMD from a SWAN visit that occurred ~2 years later. MT stage used for stratification is the stage at which each CRP and BMD pair were obtained. (Note that for stages 1 and 2, MT stage have advanced by the time of the second BMD visit). Mean change shown in bold font if mean is statistically different from zero (*p* < .05).

^d^ CRP mean change and BMD slope mean change compared across MT stages using linear regression with Huber‐White robust standard errors to account for correlations between repeated measures in the same women.

IFE regression models quantified the relation between within‐woman change in log_2_ CRP and within‐woman change in LS or FN BMD slope. Adjusted model 1 included MT stage, BMI, cigarette use, diabetes, and alcohol use. Model 2 additionally included bone beneficial medications, bone negative medications, oral corticosteroids, and NSAIDs and aspirin. Model 3 added supplemental calcium and vitamin D. In crude and adjusted models that did not include a CRP and MT stage interaction, the relation between CRP and LS BMD slope was not statistically significant (all *p* values >.2) (data not tabulated). The addition of an interaction between MT stage and CRP revealed statistically significant effect modification of the relation between within‐woman change in CRP and change in BMD slope by MT stage (omnibus interaction *p* values <.001 in all crude and adjusted models, data not tabulated). In crude and adjusted LS models with CRP and MT stage interactions, all pairwise comparisons of MT stage‐specific effects were statistically significantly different from each other (all *p* values <.03).

The relation between within‐woman change in CRP and change in FN BMD slope was statistically significant in crude and adjusted models that did not include an interaction (all *p* values <.02). Adding an interaction between MT stage and CRP to the FN models again disclosed statistically significant effect modification by MT stage (omnibus interaction *p* values <.04 in all models). In all crude and adjusted FN models, pairwise comparisons between effects in stage 1 versus 2 and stage 2 versus 3 were statistically significantly different from each other (all *p* values <.03), but pairwise comparisons between stage 1 and 3 were not (all *p* values >.6).

Because MT stage modified the relation between within woman changes in CRP and BMD slope, we proceeded to run models stratified by MT stage (Table 3). Since the exposure was log_2_ CRP, the β coefficients shown in Table 3 estimate the within‐woman change in BMD slope in relation to a within‐woman doubling of CRP. At the LS, a significant association between increasing CRP and ensuing hastening decline in BMD was evident during stage 3 (late postmenopause) only. Unadjusted, in stage 3, there was a 0.11% per year faster decline in LS BMD per CRP doubling (*p* = .002). Adjustment for all covariates did not alter this association between late postmenopausal CRP and subsequent LS BMD loss; the fully adjusted model demonstrates a 0.10% per year greater decline in LS BMD for each within‐woman doubling of CRP (*p* = .007).

**Table 3:** Associations of Within‐Woman Change in CRP and Within‐Woman Change in LS or FN BMD Slopes by MT Stage

		MT stages^a^					
		Stage 1		Stage 2		Stage 3	
Bone site	Model^b^	β (95% CI)^c^ ^,^ ^d^	*p* ^e^	β (95% CI)^c^ ^,^ ^d^	*p* ^e^	β (95% CI)^c^ ^,^ ^d^	*p* ^e^
	Crude	−0.03 (−0.08, 0.02)	.2	0.04 (−0.03, 0.11)	.3	**−0.11 (−0.18, −0.04)**	**.002**
LS BMD slope (% per year)	Adjusted 1	−0.03 (−0.08, 0.03)	.3	0.05 (−0.03, 0.12)	.2	**−0.10 (−0.17, −0.03)**	**.007**
	Adjusted 2	−0.03 (−0.08, 0.03)	.3	0.05 (−0.03, 0.12)	.2	**−0.10 (−0.17, −0.02)**	**.009**
	Adjusted 3	−0.03 (−0.08, 0.03)	.3	0.05 (−0.03, 0.12)	.2	**−0.10 (−0.17, −0.03)**	**.007**
	Crude	**−0.13 (−0.19, −0.07)**	**<.001**	−0.05 (−0.13, 0.03)	.2	**−0.15 (−0.23, −0.07)**	**<.001**
FN BMD slope (% per year)	Adjusted 1	**−0.08 (−0.15, −0.02)**	**.007**	0.01 (−0.08, 0.09)	.9	**−0.09 (−0.17, −0.01)**	**.03**
	Adjusted 2	**−0.09 (−0.15, −0.02)**	**.005**	0.00 (−0.08, 0.09)	.9	**−0.09 (−0.17, −0.00)**	**.04**
	Adjusted 3	**−0.09 (−0.15, −0.02)**	**.006**	0.00 (−0.08, 0.09)	.9	**−0.09 (−0.17, −0.01)**	**.03**

*Note*: LS BMD sample size is 1426 and the number of observations is 7618 for each model. FN BMD sample size is 1429 and the number of observations is 7628 for each model. Bold values are significant at *p* < .05.

Abbreviations: BMD, bone mineral density; CI, confidence interval; CRP, C‐reactive protein; FN, femoral neck; LS, lumbar spine; MT, menopause transition; NSAID, nonsteroidal anti‐inflammatory drug.

^a^ Stage 1 includes premenopause and early perimenopause (premenopause defined as menses unchanged from usual; early perimenopause defined as menses in prior 3 months but less predictable than usual). Stage 2 includes late perimenopause and early postmenopause (late postmenopause defined by at least 3 months but not 12 consecutive months of amenorrhea; early postmenopausal defined as the first 3 visits at which participant is postmenopausal, menopause having been determined by 12 consecutive months of amenorrhea). Stage 3 is late postmenopause, defined as the time subsequent to early postmenopause.

^b^ Covariates in adjusted model 1 are MT stage, body mass index, cigarette use, diabetes, alcohol use. Model 2 covariates include those in model 1 and bone beneficial medications, bone negative medications, oral corticosteroids, NSAIDs and aspirin. Model 3 covariates include those in models 1 and 2 and supplemental calcium and vitamin D.

^c^ β coefficients represent the change in BMD slope (annualized percent change per year in the 1.5‐year to 3.5‐year interval after CRP measurement) per doubling of CRP. A negative coefficient indicates increase in rate of BMD loss.

^d^ To obtain annualized rate of change (slope) in BMD following each CRP measure, a pair of BMDs correspond to each CRP. The first BMD was assessed simultaneously with the CRP, and therefore was measured in the specified MT stage. The second BMD of the pair was obtained up to 3.5 years later, therefore could have been measured in the same or the following MT stage.

^e^ Test that change in BMD slope is non‐zero.

At the FN BMD site, increases in CRP levels during stage 1 (premenopause and early perimenopause) and stage 3 (late postmenopause) were associated with faster subsequent BMD decline (Table 3). A doubling of CRP during stage 1 predicted a 0.13% per year faster FN BMD decline (*p* < .001). The fully adjusted model estimated a 0.09% faster yearly drop in subsequent FN BMD per CRP doubling in stage 1 (*p* = .006). Unadjusted FN models estimated that each CRP doubling during stage 3 (late postmenopause) predicted 0.15% more BMD loss per year (*p* = .03); fully adjustment diminished the effect size moderately, to −0.9% per year (*p* = .03).

## Discussion

In this longitudinal analysis of 1431 mid‐life women, within‐woman increases in CRP levels predicted increases in the rates of bone loss in the next ~2 years and this association varied by the MT stage in which the CRP was measured. In MT stage 1 (premenopause and early perimenopause), gains in CRP were related to increased rates of BMD loss only at the FN. In MT stage 2 (late perimenopause and early postmenopause), although LS and FN BMD loss occurred at even faster rates than that during MT stage 1, increases in CRP levels were unrelated to BMD declines. Finally, during stage 3 (late postmenopause), rises in CRP once again forecasted increases in BMD losses at both the LS and FN.

Previous work has not examined the relation between *change* in CRP and *change* in the rate of BMD loss as women advanced through the MT in a single, longitudinal design. In premenopausal women, sex‐specific analyses of CRP and BMD are confined to cross‐sectional approaches that yielded mixed results.^(^ ^8^, ^11^, ^13^ ^)^ Cross‐sectional studies from a Korean Health Promotion Center and from the Framingham Offspring cohort found a negative association between CRP level and BMD level in premenopause, whereas a report using baseline SWAN data observed no relation.^(^ ^8^, ^11^, ^13^ ^)^ Sex‐specific longitudinal studies in postmenopausal women describe a negative association between CRP and BMD.^(^ ^10^, ^13^ ^)^ In a Tasmanian sample of 93 women, 95% of whom were postmenopausal, an increase in CRP over an average of 2.9 years was related to a decline in total body BMD over the same interval. A Swedish cohort of 932 late menopausal women who had CRP and BMD measures at baseline and 5 years later found that those with persistently high CRP (>3 mg/L) had greater FN BMD loss during the same 10‐year interval. Access to multiple repeated measures allowed us to employ a more advanced longitudinal IFE approach, modeling individual, within‐woman change in CRP as a predictor of subsequent change in her BMD loss rate, which revealed a negative association between them during premenopause and early perimenopause and postmenopause. We did not find a link between change in CRP and BMD loss rate during late perimenopause and early postmenopause; to our knowledge, there are no prior investigations with which to compare results from this MT stage.

The current analysis detected a statistically significant association between within‐woman increases in CRP and within woman increases in BMD decline rates in MT stages 1 and 3, but the magnitude of CRP's effect was quite modest. Specifically, a 0.1% per year faster decline in BMD for each doubling of CRP is a small increase in the BMD decline rate, when taken in the context of the amount of CRP change observed and the rate at which BMD declines during this life stage in women. Within‐woman change in log_2_CRP is the exposure, and its SD over the course of the entire study was 1.31; an SD increase in the exposure variable (change in CRP) translates to a 2.5‐fold increase in CRP, and a worsening of BMD loss by 0.13% per year, which represents a modest increase over the observed mean rate of BMD decline in the cohort of 1.61% per year at the LS and 1.28% per year at the FN, during the MT's rapid phase of bone loss (stage 2).

MT stage modified the relation between change in CRP and change in BMD, but in a manner that may seem counterintuitive: There was no relation between change in CRP and change in the rate of BMD loss in late perimenopause and early postmenopause, the time when BMD declines most and when we expected to see the largest gains in CRP.^(^ ^21^ ^)^ In vitro and animal experiments led to our thesis that the exposure variable, CRP, would increase differentially in relation to the FMP, due to attenuation of estradiol's inhibitory effect on cytokine production.^(^ ^4^, ^15^, ^16^ ^)^ Because estradiol levels drop most rapidly during late perimenopause and early postmenopause, we reasoned that CRP might climb at a similarly accelerated rate. Crude mean values of CRP by stage appear concordant with that biological hypothesis: between stage 1 and stage 2, mean CRP rose from 1.42 to 1.80 mg/L (~27% increase) and remained elevated at 1.66 in stage 3 (~17% greater than stage 1). These crude means are influenced by both within‐woman and between‐woman differences, however. Scrutiny of the within‐woman change in CRP by MT stage reveals that the amount of CRP increase (relative to baseline) is constant in each MT stage, at 7.2%. In stage 2, the mean BMD decline rate is more than 1% per year, but there is only 0.05% (or less) greater annualized BMD loss per CRP doubling. Therefore, in stage 2 the CRP‐related effect is a minor fraction of the total BMD change; that is, the signal is overwhelmed by the noise (the influence of factors other than CRP). Moreover, the magnitudes of the associations between change in CRP and change in BMD slope at the LS (stage 3) and at the FN (stages 1 and 3) were quite similar, ranging from at 0.09 to 0.10 greater rate of BMD loss for each within‐woman CRP doubling. In stages 1 and 3, the BMD slope is smaller; because the CRP signal represents a bigger component of that BMD change, it is discernible.

There are two salient considerations with respect to the exposure variable, CRP: its absolute levels and the treatment of “high” CRP values. Is it plausible that our study did not find a large‐magnitude relation between change in CRP and rate of bone loss because, compared to studies that did find a CRP–BMD relation, our CRP values were lower.^(^ ^8^, ^10^, ^12^ ^)^ This was not the case; other studies’ mean CRP values ranged from 0.7 to 2.5 mg/L, with SD similar to SWAN's.^(^ ^8^, ^10^, ^12^ ^)^ Regarding “high” CRP values, we did not adopt the frequently‐used level of >10 mg/L as a definition of acute inflammation (and therefore an exclusionary criterion) because such levels can be present and persist in the absence of acute illness and are more likely to occur in subgroups.^(^ ^22^ ^)^ In the Coronary Artery Risk Development in Young Adults Study (CARDIA) study, 10% of CRP values were >10 mg/L and 3% of participants had persistent levels greater than this value. Obese women (BMI >31 kg/m^2^) accounted for 70% of repeated elevations; female sex, lower income, Black race, and hypertension were each independent predictors of persistent CRP level above 10 mg/L. In obese women, the probability that a CRP elevation was single‐time elevation reached 80% only when the cut‐point was set at 22 mg/L. Further, CRP values of 10 mg/L are not rare—they are present in 10% of all US adults and 13% of those in poverty.^(^ ^25^ ^)^ Given the frequency, persistence, and greater prevalence of CRP values of greater than 10 in many individuals and in specific population subgroups, we believe that a uniform absolute threshold for identification of acute inflammation should be supplanted by individual‐specific thresholds that account for the individual's chronic level of inflammation. In repeated measures, longitudinal data sets, this can be achieved by making comparisons of current CRP values against each individual's previous and future CRP measurements.

The main limitation of this work is that CRP was measured using three different assays over the course of SWAN, an unavoidable consequence of being in the field for over two decades. However, the SWAN laboratory cross calibrated each of three assays to a fourth, high‐sensitivity assay with excellent results. After calibration, correlations between the original three CRP assays and the fourth, high‐sensitivity ELISA were each >0.94.

The strengths of this study stem from SWAN's 17 serial waves of data collection. Multiple repeated measurements supported the use of IFE regression and a lagged exposure‐outcome design, which jointly support causal inference even within an observational study. IFE regression quantifies the relationship between *within‐woman* changes in predictor and outcome, thereby removing *all* confounding by individual‐specific stable characteristics. Specifically, IFE eliminates confounding by *time‐fixed* variables that are measured (e.g., race/ethnicity, study site) as well as those that have not or cannot be ascertained (e.g., genetics, intrauterine environment, family history).^(^ ^23^, ^24^, ^26^ ^)^ Additionally, because one would not expect bone density to change instantaneously with a rise in CRP (but, rather, to manifest after a phase of increased bone turnover in response to inflammation), we modeled change in CRP in relation to change in BMD in the following ~2‐year period. SWAN's repeated measures intentionally canvassed mid‐life and early old age, detailing the transition from premenopause to postmenopause. This design enables researchers to take a life‐course approach—the observed effect modification by MT stage underscores the importance of this capacity.

In conclusion, within‐woman increases in CRP during premenopause and early perimenopause and late postmenopause predict faster BMD decline in the next ~2 years, but the magnitude of CRP's effect on BMD is small. Thus, a change in CRP‐change in BMD pathway is unlikely to have an important influence on fracture risk in women who are similar to those studied. Nonetheless, CRP may operate as a fracture risk factor through other avenues, such as influencing bone turnover, bone size, or microarchitecture.^(^ ^11^, ^27^, ^28^ ^)^ Future work should examine the relations between change in CRP and other fracture risk factors, such as bone turnover or composite measures of bone strength, as well as the unexplored area of change in CRP and fracture risk.

## Conflict of Interest

The authors declare that no conflict of interest exists.

## AUTHOR CONTRIBUTIONS

**Gail Greendale:** Conceptualization; data curation; formal analysis; funding acquisition; investigation; project administration; resources; supervision; writing‐original draft; writing‐review & editing. **Nicholas Jackson:** Formal analysis; methodology; writing‐review & editing. **Weijuan Han:** Data curation; formal analysis; writing‐review & editing. **Mei‐Hua Huang:** Data curation; formal analysis; investigation; project administration; writing‐review & editing. **Jane Cauley:** Investigation; writing‐review & editing. **Carrie Karvonen‐Gutierrez:** Investigation; project administration; writing‐review & editing. **Arun Karlamangla:** Conceptualization; data curation; formal analysis; methodology; writing‐review & editing.

### Peer review

The peer review history for this article is available at https://publons.com/publon/10.1002/jbm4.10480.

## Data Availability

SWAN provides access to public use datasets that include data from SWAN screening, the baseline visit and follow‐up visits (https://agingresearchbiobank.nia.nih.gov/). A link to the public use datasets is also located on the SWAN web site (http://www.swanstudy.org/swan-research/data-access/). To preserve participant confidentiality, some, but not all, of the data used for this manuscript are contained in the public use datasets. Investigators who require assistance accessing the public use dataset may contact the SWAN Coordinating Center at the following email address: swanaccess@edc.pitt.edu.
